# Comparative efficacy and safety of chlorthalidone and hydrochlorothiazide—meta-analysis

**DOI:** 10.1038/s41371-019-0255-2

**Published:** 2019-10-08

**Authors:** Stela Dineva, Katya Uzunova, Velichka Pavlova, Elena Filipova, Krassimir Kalinov, Toni Vekov

**Affiliations:** 1 Science Department, Tchaikapharma High Quality Medicines, Inc, 1 G.M. Dimitrov Blvd, 1172 Sofia, Bulgaria; 2 Department of Informatics, New Bulgarian University, 21 Montevideo Str, 1618 Sofia, Bulgaria; 3 Department of Pharmacy, Medical University, Pleven, Bulgaria

**Keywords:** Hypertension, Health care

## Abstract

Hypertension is a complex syndrome of multiple hemodynamic, neuroendocrine, and metabolic abnormalities. The goals of treatment in hypertension are to optimally control high blood pressure and to reduce associated cardiovascular morbidity and mortality using the most suitable therapy available. Hydrochlorothiazide (HCTZ) and chlorthalidone (CTLD) are with proven hypertensive effects. The topic of our meta-analysis is to compare the efficacy of HCTZ and CTLD therapy in patient with hypertension. A search of electronic databases PubMed, MEDLINE, Scopus, PsyInfo, eLIBRARY.ru was performed. We chose the random-effects method for the analysis and depicted the results as forest plots. Sensitivity analyses were performed in order to evaluate the degree of significance of each study. Of the 1289 identified sources, only nine trials directly compared HCTZ and CTLD and were included in the meta-analysis. Changes in SBP lead to WMD (95% CI) equal to −3.26 mmHg showing a slight but statistically significant prevalence of CTLD. Results from analyzed studies referring to DBP lead to WMD (95% CI) equal to −2.41 mmHg, which is also statistically significant. During our analysis, we found that there were not enough studies presenting enough data on the effect of CTLD and HCTZ on levels of serum potassium and serum sodium. Our meta-analysis has demonstrated a slight superiority for CTLD regarding blood pressure control. At the same time, the two medications do not show significant differences in their safety profile.

## Introduction

Hypertension is a complex syndrome of multiple hemodynamic, neuroendocrine, and metabolic abnormalities [1]. The goals of treatment in hypertension are to optimally control high blood pressure (BP) and to reduce associated cardiovascular morbidity and mortality [2, 3]. Hypertension affects approximately one of every three adults in the United States [4] and is responsible for more than one of every eight premature deaths worldwide [5, 6].

Thiazide-type diuretics are one of the initial agents, which are used if there is no complication of hypertension and no presence of comorbid conditions in which another class of antihypertensive drug should be used [7]. List of thiazide-type diuretics include chlorthalidone (CTLD), chlorothiazide, metolazone, indapamide (INDAP), and hydrochlorothiazide (HCTZ). The most commonly prescribed antihypertensive drug of this class is HCTZ. The choice between HCTZ and CTLD for the treatment of hypertension is debatable and has lately been a topic of the science literature [8–13]. CTLD may have potentially better 24-h BP control than HCTZ [14]. The first study which implies that CTLD may be superior to HCTZ has begun in 1973, a large primary prevention trial named The Multiple Risk Factor Intervention Trial (MRFIT). In 1980, the MRFIT Policy Advisory Board recommended CTLD over HCTZ for initial hypertension therapy changing the hypertension treatment protocol [15, 16].

CTLD and HCTZ are structurally similar compounds [17]. The common element in the molecular structure of CTLD and HCTZ is the sulfonamide group, which is connected to their potential of carbonic anhydrase inhibition. However, the molecular structure of CTLD allows additional inhibition of carbonic anhydrase activity. The latter has provoked investigation of possible cardiovascular benefits since it is known to evoke cardiovascular effects and platelet function with other drug classes [18]. CTLD has longer elimination half-life than HCTZ 40–60 h compared with 6–15 h. Apart from the longer duration of action, CTLD is approximately twice as potent as HCTZ. This shows that these two compounds are quite dissimilar pharmacokinetically despite their similar structure [19, 20]. Due to BP-lowering efficacy throughout the nighttime hours half the dose of CTLD is more effective in lowering SBP than HCTZ. Differences in central BP and arterial stiffness would be postulated by persistence of BP-lowering efficacy [14, 21]. Differences in the effects of the two drugs on clinical outcomes remain unclear but evidence of benefit of low-dose thiazide-based regimens in reducing CVEs seems to be mainly derived from trials of CTLD, whereas HCTZ remains inferior to other classes of hypertensive drugs as well, including angiotensin-converting enzyme inhibitors [22] and calcium channel blockers [23]. Furthermore, a retrospective comparative analysis demonstrated that CTLD reduces CVEs more than HCTZ [7]. Therefore, a recent guideline recommended the use of CTLD or INDAP in preference to HCTZ [21, 24].

Both preparations have FDA-approved indications for the treatment of hypertension and edema. Thiazide diuretics interfere with Na+/Cl− transporter in the distal convoluted tubule and in this way prevent reabsorption of sodium and chloride [25] and probably influence electrolyte balance. The use of thiazide diuretics is commonly associated with electrolyte imbalance like hypokalemia. Comparing monotherapy of the two agents there were no statistically significant results but head-to-head trials  have shown CTLD to lower serum potassium concentration less than HCTZ [19, 20]. This contradictory evidence led us to believe that a meta-analysis evaluating the efficacy and safety of CTLD compared with HCTZ is necessary.

## Methods

The main aim of our analysis is to compare the influence of HCTZ and CTLD on systolic and diastolic BP and on the levels of serum sodium and serum potassium in patients with mild to moderate essential hypertension and to reinterpret evidence of interchangeability of HCTZ and CTLD.

### Data sources and search strategy

We searched for evidence in PubMed, Medline, Scopus, PsyInfo, eLIBRARY.ru, as well as registries for data of clinical trials (http://ClinicalTrials.gov and http://www.clinicaltrialsregister.eu) (1975–2017/Dec) using the following keywords: hydrochlorothiazide, chlortalidone, diuretics, hypertension, blood pressure, hypokalemia, hyponatremia, potassium, sodium, clinical trial, controlled, randomi*, double blind. The following search strategy was applied: diuretics AND hydrochlorothiazide OR chlorthalidone AND blood pressure OR hypertension AND hypokalemia OR potassium AND hyponatremia OR sodium AND clinical trial AND controlled AND randomized OR observational OR double blind. We searched for full-text articles and abstracts published in Latin (English) and Cyrillic. Results in Cyrillic were not found.

### Inclusion criteria

1. Randomized controlled studies and observational studies investigating different doses of CTLD and HCTZ;
2. CTLD and HCTZ alone or in combination with other antihypertensive regimen;
3. Determination of changes in systolic and/or diastolic BP and/or determination of changes in the serum levels of Na+ and/or K+;
4. Type of participants: patients with mild to moderate essential hypertension.

### Quality assessment

Effective Public Health Practice Project was utilized to assess study quality. This tool includes assessment of different characteristics like selection bias, study design, blinding, data collection method, confounders, and dropouts in order to help raters form an opinion of quality based upon information contained in the study. Mixed methods studies can be quality assessed using this tool with the quantitative component of the study. Two of the seven studies which we included in our statistical analysis: retrospective observational cohort analysis [7], retrospective analysis [26] were deemed to be of weak quality due to their minimum scores regarding questions of randomization and blinding. Table S1 for quality assessment of the included in the meta-analysis studies is presented in the Supplementary material file.

### Data extraction and statistical analysis

**Fig. 1:**
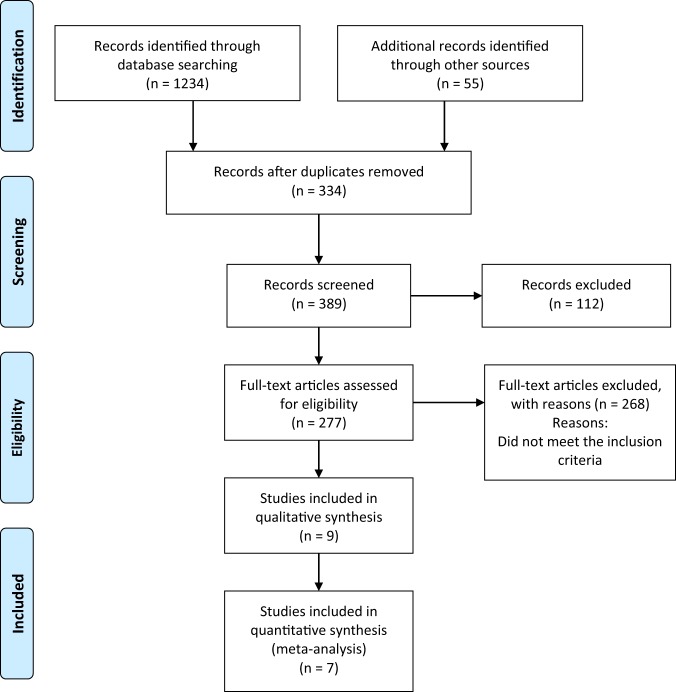
Study selection process

Identified studies were carefully reviewed, sorted, and assessed. Figure 1 presents a flow diagram that describes the process of screening of identified studies. Extraction of data was conducted by two independent reviewers and encompassed publication year, type of study, duration of treatment, number of patients, systolic BP, diastolic BP, levels of serum sodium, and levels of serum potassium. Data for all parameters were presented as weighed mean difference with a 95% confidence interval (CI). Extraction form is included in the Supplementary material file.

Due to the significant heterogeneity of the individual studies, we chose the random-effects method as the primary analysis. To assess heterogeneity of the treatment effect among trials, we used the Cochran *Q* and the *I*^2^ statistics, where *p* values of <0.10 were used as an indication of the presence of heterogeneity and an *I*^2^ parameter >50% was considered indicative of substantial heterogeneity. The threshold for statistical significance was set at 0.05.

Forest plots present estimated results from the studies included in the analysis by Weighted Mean Difference (WMD) and also we performed a sensitivity analyses in order to evaluate the degree of significance of each study. The analysis was made by subsequently excluding each study to assess its influence on the results. The calculations and graphics are made by module MetaXL (add-ins on Microsoft Excel). We also made funnel plots in order to assess publication bias and we presented them in a Supplementary material file.

## Results

The complete study selection process is shown in Fig. 1. We screened a total of 1289 articles, abstracts, and meta-analysis. Among them 1012 were excluded due to being  duplicated or unrelated to the topic, 277 proved relevant to the topic; 28 were not dealing with direct comparison between HCTZ and CTLD and only 9 [6, 7, 21, 26–30] met the inclusion criteria and were adequate for our meta-analysis. Summarized extracted data about the year of publication, duration of treatment, number of patients, and baseline SBP/DBP levels, levels of serum sodium, levels of serum potassium are presented in Table 1. The duration of trials was between 4 and 364 weeks. Two trials were observational and seven were randomized controlled. A total of 51,789 patients were included and were treated with HCTZ or CTLD as a mono- or combination therapy. HCTZ was used in the range of 12.5–100 mg/day for mono- or combination therapy, CTLD 6.25–100 mg, respectively. Patients with hypertension or with coronary heart disease of both sexes were included. Due to a great variety of doses we chose to analyze the data for most commonly used 12.5–25 mg.

**Table 1:** Characteristics of articles included in this meta-analysis

Refs	Study design	Sample size^a^	Mean baseline blood pressure (mmHg)		Mean baseline potassium (K+) (mEq/L)	Mean baseline sodium (Na+) (mEq/L)	Dose (daily)		Follow-up or treatment duration (weeks)
			Systolic	Diastolic			HCTZ	CTDN	
Bakris et al. [27]	Randomized, double-blind, double-dummy, study	609	164.6	95.4	NR/NA	NR/NA	12.5 mg + azilsartan medoxomil 40 mg)	12.5 mg (+azilsartan medoxomil 40 mg)	6
Dhalla et al. [6]	Propensity score-matched observational study	29873	NR/NA	NR/NA	>3.5	>130	12.5 mg/25 mg	12.5 mg/25 mg	260
Dorsch et al. [7]	Retrospective observational cohort analysis comparing study	6441	142.3	NR/NA	4.4	NR/NA	Individual	Individual	364
Ernst et al. [14]	Randomized, single-blinded, 8-week active treatment study	30	142.0	93.2	4.20	NR/NA	50 mg	25 mg	8
Kwon et al. [21]	Open-label, randomized, prospective cross-over study	28	152.0	94.0	4.10	143	12.5 mg (+candesartan 8 mg)	6.25 mg (+candesartan 8 mg)	4
Pareek et al. [28]	Randomized, comparative, multicenter parallel group, open-lebel study	131	152.0	95.0	4.15	139	12.5 mg (+losartan 25 mg)	6.25 mg (+losartan 25 mg)	8
Pareek et al. [29]	Double-blind, double dummy, randomized, parallel group, comparative, multicentric study	34	148.7	93.7	NR/NA	NR/NA	12.5 mg	6.25 mg	12
Saseen et al. [26]	Retrospective analysis of patients diagnosed with hypertension	856	136.2	76.9	4.04	NR/NA	25 mg	25 mg	18
van Blijderveen et al. [30]	Population-based observational case-control study	13787	NR/NA	NR/NA	NR/NA	>130	12.5 mg/25 mg	12.5 mg/25 mg	NR/NA

*NR*/*NA* not reported/not applicable

^a^The sample size includes only patients participating in the comparative analysis

### The efficacy of HCTZ therapy compared with CTLD therapy on SBP rates

Seven studies were included in this part of analysis. Three of these studies investigated the efficacy of CTLD and HCTZ in combination therapy [21, 27, 28] and four in monotherapy [7, 14, 26, 29].

Bakris et al. made a conclusion based on conducted randomized, double-blind, titrate-to-target BP trial comparing the single pill combination of azilsartan medoxomil and CTLD versus co-administration of azilsartan medoxomil and HCTZ in participants with stage 2 primary hypertension. The trial shows that CTLD combined with azilsartan medoxomil provides better BP reduction and a higher likelihood of achieving BP control than HCTZ combined with azilsartan medoxomil. This benefit occurred without a difference in safety measurements [27].

Kwon et al. conducted an open-label, randomized, prospective cross-over study which compared the antihypertensive efficacy, in combination therapy with HCTZ/candesartan versus CTLD/candesartan, on central aortic pressure. They found that CTLD, at half dose, is as potent as HCTZ (both combined with candesartan) in lowering central aortic pressure. [21].

Pareek et al. investigated the efficacy of CTLD and HCTZ in patients with mild to moderate essential hypertension. The first trial, conducted in 2009, compared the effect of CTLD and HCTZ in combination with losartan on systolic and diastolic BP [28]. In the second trial (2016) the authors compared the efficacy of CTLD and HCTZ used as monotherapies on 24-h ambulatory BP [29]. Both studies reported superiority of CTLD and explained it with its distinct pharmacokinetic profile, and its longer and smoother duration of action due to its wider volume of distribution, with partitioning into red blood cells [12, 17]. The sustained antihypertensive effects, particularly throughout the night and in the early morning hours, may be the reason for CTLD’s well-documented benefits for reduced cardiovascular morbidity and mortality [31–33]. Thus, it is not surprising that when higher-dose-outcome data were compared, CTLD proves superior to HCTZ [7, 16, 29].

Dorsch et al. performed a retrospective observational cohort study and the objective of their analysis was to evaluate the effects of CTLD and HCTZ on CVE rates. Comparing both the therapies, they reported that CTLD reduces CVEs more than HCTZ, suggesting that CTLD may be the preferred thiazide-type diuretic for hypertension in patients at high risk of CVEs [7].

Ernst et al. conducted randomized, single-blinded, 8-week active treatment, crossover study compared CTLD and HCTZ in untreated hypertensive patients. They concluded that within recommended doses, CTLD is more effective in lowering systolic BPs than HCTZ, as evidenced by 24-h ambulatory BPs. They also suggest that this finding may have a pharmacokinetic basis. Longer elimination half-life of CTLD could help sustain a prolonged low level of diuresis, resulting in lower mean nighttime BP [14].

Saseen et al. compared the clinical effectiveness and drug toxicity of CTLD and HCTZ. They made a retrospective analysis of patients diagnosed with hypertension and enrolled in a large health plan with a current membership of more than 200,000 members from 50 counties in the southern region of the United States. The health plan’s electronic health record database was used to extract data from January 1, 2005 to December 31, 2012. Their findings indicated that treatment with CTLD was associated with greater reductions in BP and higher rates of achieving goal BP values than treatment with HCTZ and in opposite of very well tolerated, drug toxicity related to metabolic adverse effects can manifest as hypokalemia, hyperglycemia, hyponatremia, and/or hyperuricemia [26].

**Fig. 2:**
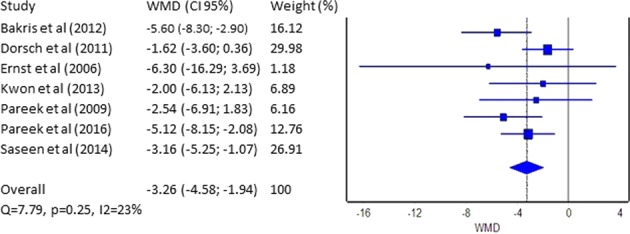
Forest plot–weighted mean difference (WMD)–SBP (mmHg)

Figure 2 shows a statistical analysis of these seven studies. The WMD (95% CI) is −3.26 mmHg (4.5 ÷ 2 mmHg). Most significant weight for our analysis has the data reported by Dorsh et al. with 30.0% and Saseen et al. with 26.9%. Cochran’s *Q* of 7.79, *p* = 0.25, and *I*^2^ = 23% (Fig. 2) signify the high degree of homogeneity of the different studies. These results support our observation for a slight superiority of CTLD. Figure S1 for publication bias using funnel plot is presented in the Supplementary material file.

### The efficacy of HCTZ therapy compared with CTLD therapy on DBP rates

**Fig. 3:**
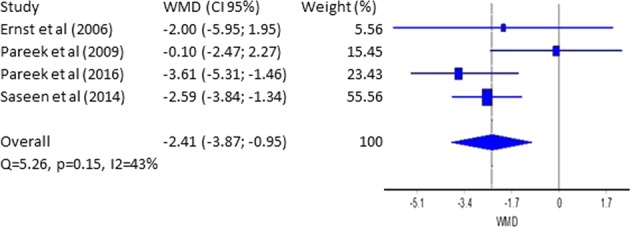
Forest plot–weighted mean difference (WMD)–DBP (mmHg)

Only four studies [14, 26, 28, 29] included data about measurements of DBP. Our statistical analysis is presented in Fig. 3. The WMD (95% CI) is −2.41 mmHg (4 ÷ 1 mmHg), and the expectation is DBP to be reduced average with 2.4 mmHg. Saseen et al. and Pareek et al. are with the most significant weight for our analysis—55.6% and 23.40%, respectively. Cochran’s *Q* of 5.26, *p* = 0.15, and *I*^2^ = 43% (Fig. 3) signify the high degree of homogeneity of the different studies. These results support our observation for the slight superiority of CTLD. Figure S2 for publication bias using funnel plot is presented in the Supplementary material file.

### Safety monitoring report

**Fig. 4:**
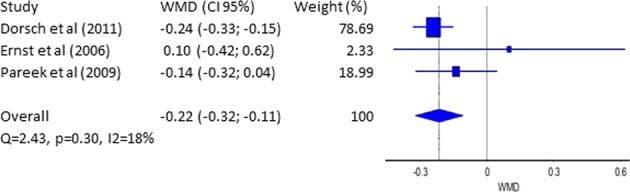
Forest plot–weighted mean difference (WMD)–serum К^+^(mEq/L)

Diuretic-related side effects can be separated into several categories, including those with well–worked-out mechanisms, such as electrolyte defects and/or metabolic abnormalities [34]. Mechanisms that contribute to the onset of hypokalemia during diuretic use include: increased flow-dependent distal nephron K+ secretion (more commonly observed with a high Na+ intake), a fall in distal tubule luminal chloride (Cl–) metabolic alkalosis, and/or secondary hyperaldosteronism [35, 36]. Sica et al. give an opinion that thiazide-related side effects are somewhat more common with longer-acting compounds, such as CTLD [34]. In our meta-analysis as a secondary point we decided to made a statistical analysis of data concerning changes of serum potassium and serum sodium levels. Safety monitoring observation of the serum potassium levels were made in three studies [7, 14, 28]. The WMD (95% CI) is −0.22 mEq/L (−0.32 ÷ −0.11 mEq/L). Dorsch et al. has the most significant weight for our analysis—78.7%. Figure 4 shows that there is no homogeneity between the published studies. No other studies included enough data about potassium levels to be included in the analysis. Figure S3 for publication bias using funnel plot is presented in the Supplementary material file.

Only one study [28] directly compared the two preparations in regard to their effects on serum sodium levels. Pareek et al. conclude that there are no significant changes in serum electrolytes, blood sugar, and other laboratory parameters in patients treated with CTLD and HCTZ.

### Sensitivity analysis

The results from the sensitivity analysis for all studies comparing efficacy of CTLD and HCTZ on SBP, using WMD as a relative association measure are presented in Table 2. It should be noted that subsequent exclusion of each study leads to pooled WMD ranging from –3.96 to –2.81, which means that there are no statistically significant differences in the reported results of all these studies.

**Table 2:** Sensitivity analysis

Excluded study	Pooled WMD (95% CI)–SBP (mmHg)	Pooled WMD (95% CI)–DBP (mmHg)	Pooled WMD (95% CI)–K^+^ (mEq/L)
Bakris et al. [27]	−2.81 (−3.99; −1.63)	NA	NA
Dorsch et al. [7]	−3.96 (−5.26; −2.67)	NA	−0.11 (−0.29; 0.06)
Ernst et al. [14]	−3.22 (−4.64; −1.81)	−2.44 (−4.22; −0.65)	−0.22 (−0.30; −0.14)
Kwon et al. [21]	−3.35 (−4.85; −1.86)	NA	NA
Pareek et al. [28]	−3.31 (−4.82; −1.79)	−2.83 (−3.85; −1.82)	−0.23 (−0.53; 0.07)
Pareek et al. [29]	−2.99 (−4.36; −1.62)	−2.04 (−3.81; −0.27)	NA
Saseen et al. [26]	−3.30 (−5.09; −1.51)	−2.19 (−4.68; 0.31)	NA

The results from the sensitivity analysis for all studies comparing efficacy of CTLD and HCTZ on DBP are presented in Table 2. In this case subsequent exclusion of each study leads to pooled WMD ranging from −2.83 to −2.04, which also means that these are no statistically significant differences in the reported results of all these studies.

The sensitivity analysis presented in Table 2 shows that subsequent exclusion of each study observed little changes of  potassium levels. It doesn’t lead to significant differences in the results, they vary within 0.2 mEq/L.

## Discussion

The main purpose of our meta-analysis was to compare the efficacy of CTLD and HCTZ used as monotherapy or in combination with other antihypertensive treatments in patients with mild to moderate essential hypertension. After systematic review of published data we used the reported results on changes in SBP, DBP, sodium, and potassium levels to compare both preparations. Table 3 summarizes our conclusions and emphasizes the superiority of CTLD when it comes to BP control.

**Table 3:** CTLD versus HCTZ—direct comparisons

Parameter	Weighted mean difference (WMD)	95% CI		Conclusion for efficacy of the preparations
		Lowest value	Highest value	
Systolic blood pressure	−3.26	−4.58	−1.94	Reduces SBP with ~3 mmHg. The WMD is statistically significant
Diastolic blood pressure	−2.41	−3.87	−0.95	Reduces DBP with ~2.41 mmHg. The WMD is statistically significant
Serum potassium (mEq/L)	−0.22	−0.32	−0.11	Reduces serum potassium levels with ~0.20 mEq/L. The WMD is statistically significant

CTLD at the doses studied is a more potent antihypertensive agent resulting in greater BP lowering and better outcomes so that recent data in the literature appear to favor it for lowering SBP more than HCTZ [14]. In previous MRFIT subgroup analysis in patients with baseline rest ECG abnormalities, CTLD group had lower potassium levels and higher uric acid levels. The authors suggest that the reason for these negative findings may again relate to the potency of CTLD and the doses used in the study [37]. Improved outcomes by CTLD may be elated to differences in pharmacokinetic and pharmacodynamics properties between the two preparations [7].

Thiazide diuretics are widely used for the management of hypertension. In recent years, it has been actively debated that there is interchangeability of thiazide-type diuretics such as HCTZ and thiazide-like diuretics including INDAP and CTLD for the treatment of hypertension [38].

Other published meta-analysis supports our statement. Ernst et al. systematically searched and identified clinical trials, from 1948 to July 2008, using HCTZ or CTLD as monotherapies. They comment that both the agents are effective antihypertensives but factors, such as ethnicity, diet, and comorbidities, may influence response to thiazides. However pooled data showed that CTLD produces statistically greater SBP reduction than HCTZ in each dose partition. Authors report that incident hypokalemia is a dichotomous end point with thresholds usually varying from 3.0 to 3.5 mEq/l depending on the study [39].

Peterzan et al. performed a dose-stratified meta-analysis and meta-regression has been used to characterize the dose-response relationships for three commonly prescribed thiazide/thiazide-like diuretics, HCTZ, CTLD and bendroflumethiazide, on BP, serum potassium, and urate. Their conclusion is in agreement with ours that HCTZ lowers systolic BP less than CTLD in the dose range (12.5–25.0 mg), which can be  explained by differences in potency rather than efficacy. Reduction of serum potassium by 0.4 mmol/L was estimated to occur at 4.2, 11.9, and 40.5 mg bendroflumethiazide, CTLD, and HCTZ, respectively [40].

Two other meta-analysis conducted in 2015 and 2017 also report findings that support our conclusions. Roush et al. identified 14 randomized trials with 883 patients comparing HCTZ with INDAP and CTLD on antihypertensive potency and metabolic effects while Liand et al. investigated the effect of the three diuretics on blood electrolyte, glucose, and total cholesterol [38, 41]. Both confirm superiority of CTLD, but only Liang et al. report lack of increase of hypokalemia.

Our meta-analysis aimed to reinterpret evidence of interchangeability of HCTZ and CTLD based on their ability to influence systolic and diastolic BP and their safety profile. We reviewed and evaluated a large number of sources and tried to base our conclusions only on trials we deemed to be of satisfactory quality (see Supplementary material). Although the large number of sources and the truly numerous patients included in the analysis are a prerequisite for reduction of bias we also used funnel plots in order to assess bias (see Supplementary material). Of all authors reporting similar findings to ours, only Roush et al. [41] reported assessment of bias while only Liang et al. [38] reported usage of a tool to assess study quality. In addition, studies we decided to include in our analysis had a longer duration of follow-up compared with those included in the analysis of Peterzan et al., Roush et al., Ernst et al., and Liang et al. [38–41] (see Summary).

Our analysis has several limitations. First of all, high quality trials investigating the efficacy of CTLD are scarce as are trials investigating changes of serum potassium and sodium levels during treatment with HCTZ and/or CTLD. Second, we have evaluated the effects of HCTZ and CTLD using data for combined doses. All studies included in our statistical analysis were conducted in the last 13 years. Some differences in the inclusion and exclusion criteria, the way of measuring BP that could contribute to a different rate of heterogeneity in the studies were avoided by sensitivity analysis.

## Conclusion

Many guidelines recommend CTLD and HCTZ as a first-line therapy for mild to moderate essential hypertension. However, HCTZ seems to be the more commonly used diuretic. Our meta-analysis has demonstrated a slight superiority for CTLD regarding BP control. At the same time, the two medications do not show significant differences in their safety profile although data concerning their effect on serum potassium and sodium is scarce. Additional studies investigating rates of hypokalemia and hyponatremia should be performed. Based on our results we think CTLD should be widely used as an alternative to HCTZ in clinical practice.

## Summary

### What is known about topic

- Thiazide-type diuretics are one of the initial agents, which are used in cases of uncomplicated hypertension.
- The choice between HCTZ and CTLD for the treatment of hypertension is debatable and has lately been a topic of the science literature.
- These two compounds are quite dissimilar pharmacokinetically despite their similar structure.
- Differences in effects of the two drugs on clinical outcomes remain unclear.

### What this study adds

- The WMD (95% CI) of −3.26 mmHg for SBP change demonstrates prevalence for CTLD on the control of SBP.
- The WMD (95% CI) is −2.41 mmHg for DBP change demonstrates slight superiority for CTLD for reduction of DBP.
- The two medications do not show significant differences in their safety profile.
- CTLD should be widely used as an alternative to HCTZ in clinical practice.

## Supplementary information

Supplementary material
