# Supplementary material for: Comparative efficacy and safety of chlorthalidone and hydrochlorothiazide—meta-analysis
Source: J Hum Hypertens. 2019 Oct 8;33(11):766–74. doi: 10.1038/s41371-019-0255-2 (PMC6892412; doi:10.1038/s41371-019-0255-2)
Supplement: Supplementary file 1 — Supplementary material [file 41371_2019_255_MOESM1_ESM.docx]

**Funnel plots**

Asymmetry in funnel plots may indicate publication bias in meta-analysis, but the shape of the plot in the absence of bias depends on the choice of axes. We evaluated standard error, precision (inverse of standard error), variance, inverse of variance, sample size and log sample size (vertical axis) and log odds ratio, log risk ratio and risk difference (horizontal axis). Standard error is likely to be the best choice for the vertical axis: the expected shape in the absence of bias corresponds to a symmetrical funnel, straight lines to indicate 95% confidence intervals can be included and the plot emphasizes smaller studies which are more prone to bias. (Sterne JA, Egger M J, Funnel plots for detecting bias in meta-analysis: guidelines on choice of axis, Clin Epidemiol. 2001 Oct;54(10):1046-55.)

Figure S1: Funnel plot: Weighted Mean Difference (WMD) – SBP (mmHg).

Funnel plot including all studies shows that the results are relatively homogeneous p=0.25.


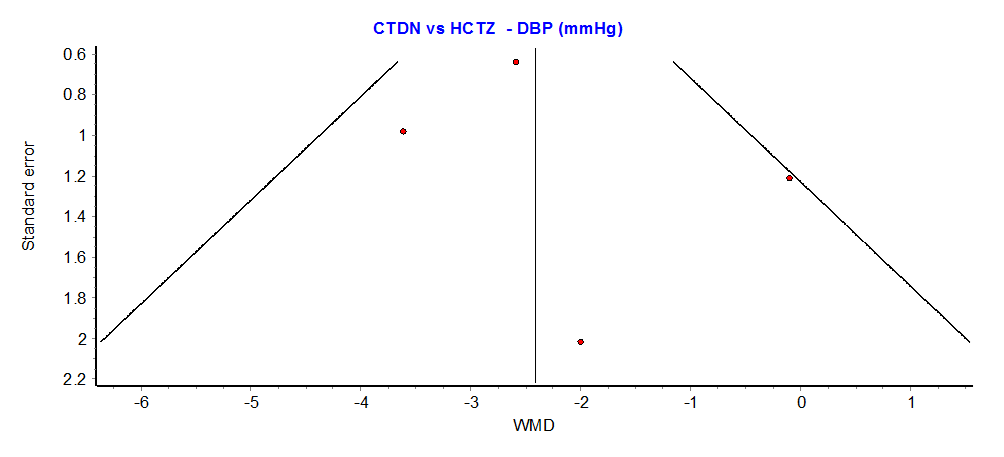


Figure S2: Funnel plot: Weighted Mean Difference (WMD) – DBP (mmHg)

Funnel plot including all studies shows that the results are relatively homogeneous p=0.15

Figure S3: Funnel plot: Weighted Mean Difference (WMD) – serum К^+^ (mEq/L)

Funnel plot including all studies shows that the results are relatively homogeneous p=0.30

**Quality assessment**

Table S1: Effective Public Health Practice Project (EPHPP) was utilized to assess study quality

| **Ref** | **Study** | **A** | **B** | **C** | **D** | **E** | **F** | **Global rating** |
| --- | --- | --- | --- | --- | --- | --- | --- | --- |
| 26 | Bakris et al (2012) | 2 | 1 | 1 | 1 | 1 | 1 | strong |
| 6 | Dhalla et al (2013) | 2 | 2 | 1 | 3 | 2 | NA | weak |
| 7 | Dorsch et al (2011) | 2 | 2 | 1 | 3 | 2 | NA | weak |
| 14 | Ernst et al (2006) | 1 | 1 | 1 | 2 | 2 | 1 | moderate |
| 21 | Kwon et al (2013) | 1 | 1 | 1 | 3 | 2 | 1 | moderate |
| 27 | Pareek et al (2009) | 1 | 1 | 1 | 3 | 2 | 1 | moderate |
| 28 | Pareek et al (2016) | 1 | 1 | 1 | 1 | 1 | 3 | moderate |
| 29 | Saseen et al (2014) | 2 | 2 | 1 | 3 | 2 | NA | weak |
| 30 | van Blijderveen et al (2014) | 2 | 2 | 1 | 3 | 2 | NA | weak |

A – Selection bias; B – Study design; C – Confounders; D – Blinding; E – Data collection method; F – Withdrawals and drop – outs; 1- Strong; 2 – Moderate; 3 – Weak; NA – Not Applicable

Two of the seven studies which we included in our statistical analysis: retrospective observational cohort analysis (7), retrospective analysis (29), we assessed like weak due to they received minimum scores regards of the questions of randomization and blinding. They are not applicable for this kind of studies. (6) and (30) are not included in the statistical analysis.
